# Pulse Wave Velocity in Atrial Fibrillation: A Scoping Review of Clinical Relevance

**DOI:** 10.7759/cureus.108092

**Published:** 2026-05-01

**Authors:** Oren G Nedjar, Zaneh Kahook, Syed M Shah, Christos G Mihos, Marc M Kesselman

**Affiliations:** 1 Internal Medicine, Nova Southeastern University Dr. Kiran C. Patel College of Osteopathic Medicine, Fort Lauderdale, USA; 2 Neurology, Nova Southeastern University Dr. Kiran C. Patel College of Osteopathic Medicine, Fort Lauderdale , USA; 3 Neuroscience, Kansas City University College of Osteopathic Medicine, Kansas City, USA; 4 Cardiology, Mount Sinai Heart Institute, Columbia University College of Physicians and Surgeons, Miami Beach, USA; 5 Rheumatology, Nova Southeastern University Dr. Kiran C. Patel College of Osteopathic Medicine, Fort Lauderdale, USA

**Keywords:** arterial stiffness, atrial fibrillation (af), atrial fibrillation recurrence, diastolic dysfunction, incident atrial fibrillation, post-operative atrial fibrillation, pulse wave velocity (pwv)

## Abstract

Atrial fibrillation (AF) is the most common sustained cardiac arrhythmia and is associated with high morbidity, mortality, and healthcare burden. Arterial stiffness (AS) alters cardiac loading conditions and contributes to ventricular arterial uncoupling (VAC), diastolic dysfunction, and atrial remodeling. Pulse wave velocity (PWV) is the standard noninvasive measure of AS, but its role across the spectrum of AF has not been clearly defined. This scoping review assessed the existing literature on PWV and AF, with attention to disease onset, clinical characteristics, progression, treatment response, and outcomes. A scoping review was conducted following Preferred Reporting Items for Systematic reviews and Meta-Analyses extension for Scoping Reviews (PRISMA ScR) guidelines.

The databases of MEDLINE, EMBASE, Web of Science, and CINAHL were searched for studies published between 2014 and 2024. Eligible studies included adult human populations with AF and reporting of at least one PWV measurement. Systematic reviews, meta-analyses, case reports, animal studies, and abstract-only publications were excluded. Study selection and data extraction were performed independently by multiple reviewers, and 26 studies met the inclusion criteria.

Most studies reported an association between higher PWV and AF, particularly in relation to atrial remodeling, adverse clinical outcomes, and postoperative AF. Central measures of AS showed more consistent associations than peripheral indices. Evidence for PWV as a predictor of new-onset AF was mixed after adjustment for traditional risk factors. Several studies suggested greater clinical relevance in patients with established AF, where higher PWV was linked to worse outcomes and increased disease burden. Measurement methods and study populations varied substantially.

Overall, the current evidence supports a meaningful relationship between AS and AF. Pulse wave velocity appears most informative as a marker of disease severity, hemodynamic stress, and clinical prognosis rather than as a standalone predictor of AF onset. Differences in PWV methodology and study design limit cross-study comparability and causal inference. Future longitudinal studies using standardized, AF-specific PWV protocols should test whether PWV improves prognostic stratification, perioperative risk assessment, and population-level prediction of AF onset.

## Introduction and background

Atrial fibrillation (AF) is the most common sustained cardiac arrhythmia in adults and represents a growing public health burden worldwide, with a global prevalence exceeding 50 million individuals in 2021 [1]. It is associated with substantial morbidity and mortality, including increased risks of stroke, heart failure (HF), myocardial infarction (MI), cognitive impairment, chronic kidney disease, and death [1-3]. 

Atrial fibrillation arises from the interplay between focal electrical triggers and a vulnerable atrial substrate shaped by progressive remodeling [4,5]. Current understanding emphasizes atrial cardiomyopathy, which encompasses alterations in atrial structure, mechanics, and electrophysiology that promote AF susceptibility and persistence [4,6]. Structural remodeling includes fibrosis, inflammation, and abnormal calcium handling, leading to nonuniform electrical propagation (conduction heterogeneity) that facilitates re-entrant circuits [4,7]. Atrial fibrosis represents a central pathological feature and is further amplified by mechanical stretch and neurohormonal activation, particularly through renin-angiotensin-aldosterone system pathways [4,8].

Arterial stiffness (AS) is a hallmark of vascular aging and reflects cumulative exposure to mechanical stress and vascular remodeling, with strong associations to cardiovascular (CV) risk and adverse outcomes [9-11]. Pulse wave velocity (PWV) is widely accepted as the gold standard noninvasive measure of AS and reflects the speed at which the pressure wave propagates along the arterial tree [10,12]. In compliant arteries, elastic expansion of the vessel wall absorbs part of the pulsatile energy and slows wave propagation, whereas increased AS limits this buffering capacity and accelerates pulse transmission [12]. Accordingly, higher PWV indicates reduced arterial compliance and is consistently associated with increased risk of CV events and mortality, supporting its clinical relevance beyond a purely physiological measurement [9,11,13]. 

The mechanistic link between AS and AF is supported by the hemodynamic consequences of impaired ventricular-arterial coupling (VAC) [10,12,14]. Increased AS accelerates PWV, resulting in an earlier return of reflected pressure waves during systole rather than diastole [10,12]. This early wave reflection augments left ventricular (LV) systolic load and wall stress, prolongs ejection, and impairs active ventricular relaxation, leading to diastolic dysfunction even in the absence of overt systolic impairment [12,14]. Elevated LV filling pressures are subsequently transmitted to the left atrium, producing chronic atrial stretch and increased wall stress that promote atrial enlargement and structural remodeling characterized by fibrosis, myocyte hypertrophy, and abnormal calcium handling [4,6,7]. These changes impair left atrial (LA) reservoir, conduit, and booster pump function and disrupt atrial electrical conduction, resulting in conduction heterogeneity and stabilization of re-entrant circuits that create a substrate permissive to AF [4,5,7]. Observational studies demonstrating associations between increased AS and impaired LA structure and phasic function, even prior to the development of overt CV disease, further support AS as an upstream contributor to atrial cardiomyopathy and AF susceptibility [15-17].

Despite increasing interest in the relationship between AS and AF, important knowledge gaps remain. Existing reviews are largely narrative and do not systematically map study designs, populations, or PWV measurement approaches, limiting their ability to synthesize the breadth of available evidence [18,19]. The present review was designed to consolidate the published data on the utility of PWV in assessing AF burden, progression, perioperative risk, treatment response, and prognosis, in addition to a reappraisal of its role in incident AF prediction.

## Review

Methods

Search Strategy

**Figure 1:**
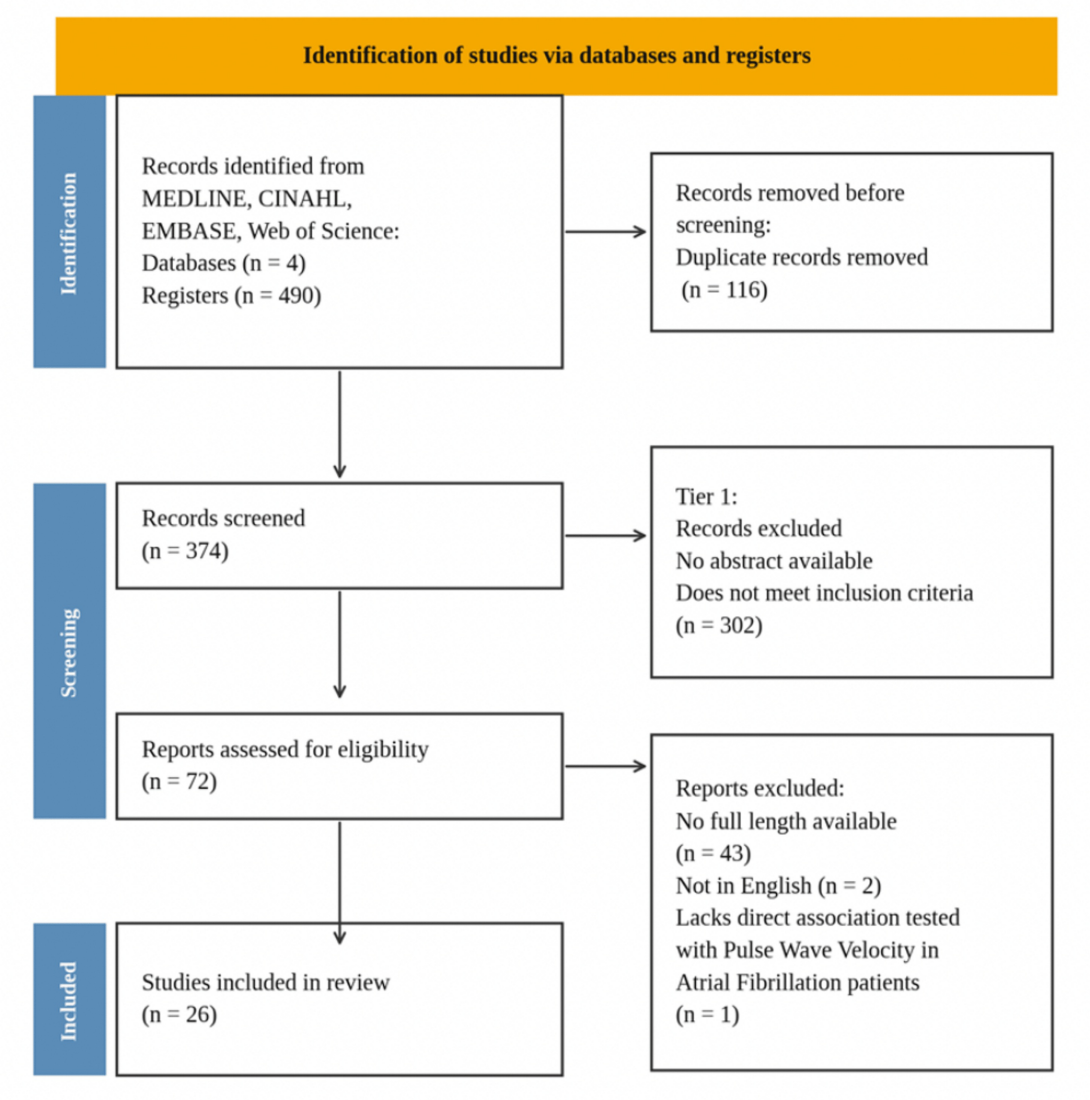
The PRISMA ScR flowchart PRISMA ScR: Preferred Reporting Items for Systematic reviews and Meta-Analyses extension for Scoping Reviews

A search strategy was built by analyzing key terms, and Boolean searches were carried out using EMBASE, MEDLINE, Web of Science, and CINAHL. Data collection took place in April 2025 using the same search process for each database. The search terms 'PWV' and 'AF' were entered into the controlled descriptors for each database. The search strategy was peer-reviewed by a librarian using the Peer Review of Electronic Search Strategies (PRESS) checklist [20]. The details of the search terms and the whole search strategy can be found in Appendix A. To be eligible for inclusion, articles were required to be written in English, peer-reviewed, published between January 1, 2014, and January 31, 2024, and involve human adults aged 18 years or older. Studies also had to include patients diagnosed with AF and report at least one measure of PWV, without restriction on the PWV modality used. Eligible study designs were limited to primary research articles and therefore had to differ from the prespecified exclusion categories. Articles were excluded if they were systematic reviews, scoping reviews, case reports, animal studies, meta-analyses, or abstracts without full-text availability. The Preferred Reporting Items for Systematic Reviews and Meta-Analyses extension for Scoping Reviews (PRISMA ScR) [21] flowchart method was utilized to streamline the inclusion process of the studies (Figure 1).

Selection of Sources of Evidence

The search identified 490 citations, limited to studies published between 2014 and 2024, as outlined in Figure 1. After removing 116 duplicate records using the Rayyan automatic software (Rayyan Systems Inc., Cambridge, MA, USA), 374 unique studies remained for screening. For tier one screening, the remaining studies were sorted alphabetically by title and independently reviewed by three reviewers. All reviewers independently screened titles and abstracts using pre-established inclusion and exclusion criteria. Discrepancies in eligibility decisions were resolved through discussion, with adjudication by a third independent reviewer when necessary. Following tier one screening, 72 articles were identified as potentially relevant for further evaluation.

For tier two screening, 43 records were excluded due to the absence of full-text manuscripts, two for non-English language, and two for lack of appropriate methodology, as documented in the PRISMA flow diagram. This resulted in the final inclusion of 26 studies. The quality and methodological rigor of the included studies were central to the selection process. All included studies underwent critical appraisal using the Joanna Briggs Institute (JBI) Critical Appraisal Tools to assess risk of bias [22]. All studies met the appraisal criteria and were retained for the final scoping review, supporting an in-depth evaluation of study quality and relevance.

Rayyan and Microsoft™ Excel (Microsoft Corp., Redmond, WA, USA) were used to manage and organize the screening and data extraction processes. These tools facilitated documentation of inclusion and exclusion decisions, participant characteristics, study context and concepts, research methods, and key findings relevant to the review. Data from Rayyan and Excel were subsequently used to complete the PRISMA flow diagram in accordance with established inclusion and exclusion criteria.

Results

PWV as a Predictor of Incident AF

Several large prospective and population-based studies evaluated PWV as a predictor of incident or new-onset AF (NOAF). In the Kailuan Study (n = 49,872), higher brachial-ankle PWV (baPWV) was independently associated with increased risk of NOAF. Participants in the highest AS quartile exhibited approximately a two-fold higher hazard of developing AF compared with those in the lowest quartile (adjusted HR ≈ 2.0, p < 0.001), with particularly pronounced associations in non-hypertensive individuals and those with higher BMI [23]. Similarly, in a large Chinese cohort followed for 11 years (n = 96,561), estimated PWV (ePWV) was a significant predictor of incident AF, with each 1 m/s increase in ePWV associated with a 14% higher risk of AF (hazard ratio (HR) 1.14, 95% CI 1.10-1.18, p < 0.001), and individuals in the highest ePWV quartile demonstrating markedly higher cumulative incidence of AF compared with those in the lowest quartile [24].

Interestingly, a prospective cohort study demonstrated a U-shaped relationship between carotid-femoral PWV (cfPWV) and incident AF. Risk was elevated in both low and high stiffness quartiles compared with intermediate levels (HR range 1.49-1.59 across extreme quartiles), demonstrating that central AS specifically was associated with AF risk [25]. In pooled analysis across three major population-based cohorts (Atherosclerosis Risk in Communities (ARIC) study, Multi-Ethnic Study of Atherosclerosis (MESA), and the Rotterdam Study; combined n > 25,000), higher aortic PWV (aPWV) was independently associated with incident AF even after adjustment for carotid intima-media thickness, with an adjusted HR of approximately 1.2 to 1.3 per standard deviation increase in aPWV, indicating that the association between AS and AF incidence was independent of structural atherosclerosis [26].

In patients with systolic heart failure (n = 77), higher baseline aPWV was associated with increased incidence of NOAF during follow-up. Patients who developed AF exhibited significantly higher baseline aPWV compared with those who did not (7.1 ± 2.6 vs 5.3 ± 1.9 m/s, p = 0.004) [27]. In contrast, findings from the Framingham Heart Study (n = 5,797) demonstrated that cfPWV was not independently associated with incident AF after full multivariable adjustment for CV risk factors (p = 0.18), although other vascular measures including augmentation index (HR 1.16, 95% CI 1.02-1.32, p=0.02), central pulse pressure (HR 1.14, p=0.02), and endothelial dysfunction (lower flow-mediated dilation: HR 0.79, p=0.04) remained significantly associated with AF risk [28]. Similarly, in a population-based cohort examining CV outcomes (n = 1,872), cfPWV was not independently associated with the composite outcome of heart failure hospitalization or AF after adjustment for confounding variables (p > 0.05) [29].

Association Between PWV and Presence or Type of Established AF

Multiple cross-sectional and case-control studies examined whether PWV differed between patients with established AF and control populations, as well as across AF subtypes. In a cross-sectional case-control study (n = 151), patients with paroxysmal and persistent AF exhibited significantly higher cfPWV compared with age-matched controls (8.0 vs 7.2 m/s, p < 0.001), suggesting increased central AS in individuals with AF [30]. Among hypertensive patients (n = 268), baPWV was significantly higher in those with AF compared with those without AF (1945 ± 477 vs 1695 ± 384 cm/s, p < 0.001). Furthermore, patients with persistent AF demonstrated higher baPWV than those with paroxysmal AF (p < 0.05), indicating a potential relationship between AS and AF chronicity [31].

In patients with paroxysmal AF (n = 108), cfPWV was significantly elevated compared with healthy controls (p < 0.05) and was accompanied by impaired ventricular-arterial coupling, reflecting early structural and functional CV remodeling [32]. In contrast, among patients with heart failure with preserved ejection fraction (HFpEF; n = 52), PWV did not differ significantly between those with permanent AF and those in sinus rhythm (p = 0.52), suggesting that the association between AS and AF may be context-dependent in advanced cardiometabolic disease, where overlapping processes such as left atrial myopathy, altered filling pressures, and shared cardiometabolic risk factors may play a more dominant role than AS alone [33]. In patients experiencing a first episode of nonvalvular AF (n = 34), PWV values at baseline and at 12-month follow-up were not significantly different from those observed in healthy controls (10.2 ± 2.5 vs 9.7 ± 2.1 m/s, p = 0.37), indicating that AS may not be elevated in early or isolated AF presentations [34].

In a retrospective study of ischemic stroke patients (n = 2,738), individuals classified as having early vascular aging based on elevated baPWV relative to age demonstrated a significantly higher prevalence of AF compared with those with normal vascular aging profiles (35.8% vs. 19.7%, p < 0.001) [35].

PWV and AF Burden, Recurrence, or Inducibility

Several studies assessed the relationship between PWV and AF burden, recurrence, or inducibility rather than simple AF presence. In a prospective clinical study of patients with paroxysmal AF (n = 104), higher aPWV was significantly associated with more frequent AF recurrences, with an aPWV threshold >10.0 m/s emerging as a strong independent predictor of recurrence frequency (p < 0.001) [36]. In contrast, among patients undergoing pulmonary vein isolation for paroxysmal AF (n = 44), cfPWV did not differ significantly between those who experienced AF recurrence and those who remained in sinus rhythm during follow-up (p = 0.91), indicating no association between AS and post-ablation recurrence [37].

In an electrophysiology laboratory study (n = 87), cfPWV was not associated with AF inducibility. Instead, endothelial dysfunction, as assessed by flow-mediated dilation (FMD), was the primary predictor of induced AF (OR 0.853 per unit decrease in FMD), while AS did not significantly differ between inducible and non-inducible patients [38].

PWV in Postoperative AF and Procedure-Related AF

Pulse wave velocity was also evaluated as a predictor of AF in postoperative and procedure-related settings. In patients undergoing off-pump coronary artery bypass grafting (n = 164), elevated baPWV (>19 m/s) in combination with elevated LV filling pressure (E/e′ >15) was strongly associated with the development of postoperative AF (POAF). The combined presence of both abnormalities was associated with a markedly increased risk of POAF (adjusted OR 12.5, 95% CI 2.5-63.8, p = 0.002), whereas elevated baPWV or elevated E/e′ alone was not sufficient to predict POAF [39]. Similarly, in a prospective observational study (n = 110), patients who developed POAF exhibited significantly higher aortic PWV compared with those who did not (9.4 ± 1.2 vs 8.6 ± 1.3 m/s, p = 0.006), with an aPWV threshold >9.5 m/s demonstrating modest discriminative ability for POAF (AUC 0.668) [40].

Prognostic and Cerebrovascular Implications of PWV in Patients With Established AF

Several studies examined PWV as a prognostic marker within populations of patients with established AF. In a model of acute intravascular volume expansion, higher baseline cfPWV predicted short-term adverse outcomes, including NOAF [41]. In patients with AF (n = 167), higher baPWV independently predicted adverse CV outcomes, including stroke and all-cause mortality. Each standard deviation increase in baPWV was associated with approximately 15% higher risk of adverse events (adjusted HR 1.152, 95% CI 1.054-1.259, p = 0.002), and the association was consistent across AF subtypes [42]. In AF-related stroke patients (n = 30), cfPWV demonstrated a strong positive correlation with the CHA₂DS₂-VASc (congestive heart failure, hypertension, age, diabetes mellitus, stroke, vascular disease, age, and sex) score (r = 0.672, p < 0.001), indicating that greater AS was associated with higher thromboembolic risk profiles [43].

In patients with AF undergoing transesophageal echocardiography (n = 99), higher PWV was significantly associated with more advanced aortic atherosclerosis, including increased intima-media thickness and plaque burden (p < 0.0001) [44]. Additionally, in a stroke cohort characterized by early vascular aging, AF prevalence was significantly higher among patients with elevated baPWV, further supporting an association between AS, cerebrovascular disease, and AF [35].

Reliability, Measurement Validity, and Modifiability of PWV in AF

Several studies addressed whether PWV measurements are reliable in the presence of AF and whether AS is modifiable by rhythm control or pharmacologic interventions. The PWV measurements obtained during AF were slightly higher and more variable compared with measurements during sinus rhythm following cardioversion. For cfPWV, mean values were higher during AF than sinus rhythm (9.3 ± 1.8 vs. 8.5 ± 1.6 m/s, p < 0.001), although single-site PWV measurements (not requiring two arterial recording sites) remained comparable between rhythms [45].

In a proof-of-concept interventional study (n = 34), cfPWV decreased significantly following successful cardioversion from AF to sinus rhythm (11.8 to 10.7 m/s), and PWV measurements demonstrated excellent reproducibility before and after cardioversion (intraclass correlation coefficient 0.89), supporting the reliability of PWV assessment in AF [46]. In a longitudinal study evaluating anticoagulation therapy (n = 21), switching from warfarin to rivaroxaban was associated with significant reductions in both augmentation index and baPWV after six months (p = 0.03) [47]. Similarly, in a multi-center randomized controlled trial involving hemodialysis patients with AF (n = 132), PWV changes over 18 months did not differ significantly between anticoagulation treatment strategies (p = 0.29), indicating no measurable effect of anticoagulant choice on AS in advanced vascular disease states [48].

For reference, Table 1 summarizes the quality assessment of all included studies, including study design, the specific JBI critical appraisal checklist used, and the overall quality rating. Table 2 further summarizes all included studies according to AF subtype, PWV measurement modality, sample size, summary statistics, study design, and key findings. 

**Table 1:** Quality assessment of included studies using study design-specific JBI critical appraisal tools JBI: Joanna Briggs Institute [22]

Study	Study design	Assessment tool used	Overall quality
Lundwall et al., 2024 [45]	Quasi-experimental study (pre-post within-subject design)	JBI critical appraisal checklist for quasi-experimental studies	7/9; high quality
Song et al., 2024 [23]	Prospective observational cohort study	JBI critical appraisal checklist for cohort studies	9/11; high quality
Almuwaqqat et al., 2021 [25]	Prospective cohort	JBI critical appraisal checklist for cohort studies	9/11; high quality
Chen et al., 2016 [42]	Prospective cohort study	JBI critical appraisal checklist for cohort studies	9/11; high quality
Durak et al., 2024 [38]	Cross-sectional study	JBI critical appraisal checklist for analytical cross-sectional studies	6/8; moderate quality
Pauklin et al., 2021 [30]	Analytical cross-sectional study	JBI critical appraisal checklist for analytical cross-sectional studies	8/8; high quality
Chen et al., 2016 [26]	Prospective cohort study	JBI critical appraisal checklist for cohort studies	9/11; high quality
Choi et al., 2022 [39]	Retrospective cohort	JBI critical appraisal checklist for cohort studies	10/11; high quality
Apaydin et al., 2023 [40]	Prospective cohort study	JBI critical appraisal checklist for cohort studies	9/11; high quality
Han et al., 2024 [35]	Retrospective observational cohort study	JBI critical appraisal checklist for cohort studies	9/11; high quality
Bonapace et al., 2016 [27]	Prospective observational cohort study	JBI critical appraisal checklist for cohort studies	9/11; high quality
Namba et al., 2017 [47]	Prospective pilot interventional (single-arm before-and-after)	JBI critical appraisal checklist for quasi-experimental studies	7/9; moderate-to-high quality
Chen et al., 2022 [24]	Prospective cohort study	JBI critical appraisal checklist for cohort studies	9/11; high quality
Shi et al., 2016 [31]	Cross-sectional study	JBI critical appraisal checklist for analytical cross-sectional studies	7/8; high quality
Bosanac et al., 2022 [33]	Cross-sectional study	JBI critical appraisal checklist for analytical cross-sectional studies	6/8; moderate quality
Kizilirmak et al., 2015 [36]	Analytical cross-sectional study	JBI critical appraisal checklist for analytical cross-sectional studies	8/8; high quality
Kilicgedik et al., 2017 [34]	Prospective cohort study	JBI critical appraisal checklist for cohort studies	8/11; moderate-to-high quality
Caluwe et al., 2018 [46]	Quasi-experimental study (before-and-after within-subject design)	JBI critical appraisal checklist for quasi-experimental studies	7/9; moderate-to-high quality
De Vriese et al., 2020 [48]	Randomized controlled trial	JBI critical appraisal checklist for randomized controlled trials	10/13; high quality
Frary et al., 2024 [29]	Prospective cohort study	JBI critical appraisal checklist for cohort studies	9/11; high quality
Milan et al., 2020 [41]	Prospective cohort study	JBI critical appraisal checklist for cohort studies	9/11; high quality
Szmigielski et al., 2016 [44]	Cross-sectional study	JBI critical appraisal checklist for analytical cross-sectional studies	7/8; high quality
Akkaya et al., 2023 [43]	Cross-sectional study	JBI critical appraisal checklist for analytical cross-sectional studies	6/8; moderate quality
Shaikh et al., 2016 [28]	Prospective cohort study	JBI critical appraisal checklist for cohort studies	9/11; high quality
Gaczol et al., 2024 [37]	Prospective cohort study	JBI critical appraisal checklist for cohort studies	8/11; moderate-to-high quality
Gaczol et al., 2023 [32]	Cross-sectional study	JBI critical appraisal checklist for analytical cross-sectional studies	7/8; high quality

**Table 2:** Studies included in the review AF: Atrial fibrillation; AS: Arterial stiffness; NOAF: New-onset atrial fibrillation; CV: Cardiovascular; POAF: Postoperative AF; PWV: Pulse wave velocity; cfPWV: Carotid-femoral PWV; aPWV: Aortic PWV; baPWV: Brachial-ankle PWV; faPWV: Femoral-ankle PWV; ePWV: Estimated PWV; CABG: Coronary artery bypass grafting; HF: Heart failure; HFpEF: Heart failure with preserved ejection fraction; LA: Left atrial; LV: Left ventricular; SR: Sinus rhythm; FMD: Flow-mediated dilation; E/e′: Ratio of early mitral inflow velocity to mitral annular early diastolic velocity; CHA₂DS₂-VASc: Congestive heart failure, hypertension, age ≥75 years, diabetes mellitus, stroke or transient ischemic attack, vascular disease, age 65-74 years, sex category score; HR: Hazard ratio; ICC: Intraclass correlation coefficient; AUC: area under the curve; PP: Pulse pressure

Reference	Title	Type of AF studied	Type of PWV studied	Sample size, age	Relevant study findings	Study type
Lundwall et al. 2024 [45]	Assessment of aortic stiffness during AF: Solutions and considerations	Measurement reliability in AF vs. sinus rhythm	cfPWV, single-site PWV	N=34; mean age 68±9 years	cfPWV higher during AF than sinus rhythm (9.3±1.8 vs 8.5±1.6 m/s, p0.001); single-site PWV comparable between rhythms	Quasi-experimental study (pre-post within-subject design)
Song et al. 2024 [23]	Association between brachial ankle PWV and NOAF: Kailuan study	Incident AF	baPWV	N=49,872; mean age 51.6±12.4 years	Highest quartile vs lowest: HR ~2.0 (p0.001); association stronger in non-hypertensive individuals and higher BMI	Prospective cohort
Almuwaqqat et al. 2021 [25]	Association of AS with incident atrial fibrillation	Incident AF	cfPWV	N=3,882; mean age 75±5 years	U-shaped association: HR 1.49-1.59 for extreme quartiles vs. second quartile; median follow-up 5.5 years	Prospective cohort
Chen et al. 2016 [42]	Association of brachial ankle PWV with CV events in AF	Prognostic outcomes in established AF	baPWV	N=167; mean age 71±11 years	baPWV independently predicted CV events (HR 1.152 per SD, 95% CI 1.054-1.259, p=0.002); median follow-up 26 months	Prospective cohort
Durak et al. 2024 [38]	Association of induced AF with endothelial dysfunction	AF inducibility	cfPWV	N=87; mean age 56±11 years	cfPWV not associated with AF inducibility; endothelial dysfunction (FMD) was the primary predictor (OR 0.853)	Cross-sectional
Pauklin et al. 2021 [30]	AF is associated with increased central blood pressure and AS	Established AF (paroxysmal and persistent)	cfPWV	N=151; age-matched (mean 62±9 years)	cfPWV higher in AF patients vs controls (8.0 vs 7.2 m/s, p<0.001)	Cross-sectional
Chen et al. 2016 [26]	Carotid intima-media thickness and AS, and the risk of AF	Incident AF	aPWV	N>25,000 (ARIC n=13,907; MESA n=6,640; Rotterdam n=5,220)	aPWV is independently associated with incident AF (HR ~1.2-1.3 per SD) independent of carotid IMT	Prospective cohort (pooled analysis)
Choi et al. 2022 [39]	Combined impact of elevated AS and LV filling pressure on outcomes after off-pump coronary artery bypass graft	Postoperative AF	baPWV	N=164; mean age 65±9 years	Combined baPWV >19 m/s + E/e′ >15 strongly predicted POAF (OR 12.5, 95% CI 2.5-63.8, p=0.002)	Retrospective cohort
Apaydin et al. 2023 [40]	Could we predict POAF with a simple ambulatory oscillometry evaluating aortic stiffness?	Postoperative AF	aPWV	N=110; mean age 62±9 years	Higher aPWV in POAF patients (9.4±1.2 vs 8.6±1.3 m/s, p=0.006); aPWV >9.5 m/s had AUC 0.668	Prospective cohort
Han et al. 2024 [35]	Early vascular aging determined by baPWV and ischemic stroke outcome	AF prevalence in stroke patients	baPWV	N=2,738; median age 68 years	Early vascular aging associated with higher AF prevalence (35.8% vs 19.7%, p0.001)	Retrospective cohort
Bonapace et al. 2016 [27]	Echocardiographically derived PWV and diastolic dysfunction associated with increased AF incidence	Incident AF in systolic HF	aPWV (echo-derived)	N=77; mean age 63±9 years	Higher aPWV in patients who developed AF (7.1±2.6 vs 5.3±1.9 m/s, p=0.004)	Prospective cohort
Namba et al. 2017 [47]	Effects of switching from warfarin to rivaroxaban on AS	Modifiability of PWV in AF	baPWV	N=21; mean age 72±8 years	Switching to rivaroxaban reduced augmentation index and baPWV at six months (p=0.03)	Prospective pilot interventional (single-arm before-and-after)
Chen et al. 2022 [24]	Estimated PWV predicts NOAF	Incident AF	ePWV	N=96,561; mean age 50.8±12.3 years; 11-year follow-up	Each 1 m/s increase in ePWV: HR 1.14 (95% CI 1.10-1.18, p0.001); the highest vs. the lowest quartile showed markedly higher cumulative incidence	Prospective cohort
Shi et al. 2016 [31]	Factors influencing AS in elderly hypertensive patients with AF	Established AF in hypertensive patients	baPWV	N=268; mean age 75±7 years	baPWV higher in AF vs no AF (1945±477 vs 1695±384 cm/s, p0.001); persistent AF had higher baPWV than paroxysmal (p0.05)	Cross-sectional
Bosanac et al. 2022 [33]	HFpEF and AF: Vascular and metabolic interplay	Established AF in HFpEF	PWV (type not specified)	N=52; HFpEF patients	No significant PWV difference between permanent AF and sinus rhythm (p=0.52)	Cross-sectional
Kizilirmak et al. 2015 [34]	Impact of aortic stiffness on paroxysmal AF recurrence	AF recurrence	aPWV	N=104; paroxysmal AF patients	aPWV >10.0 m/s strong predictor of AF recurrence frequency (p0.001)	Cross-sectional
Kilicgedik et al. 2017 [34]	Left atrial mechanical function and aortic stiffness in first-episode AF	First-episode AF	PWV (type not specified)	N=34; mean age 54±12 years	PWV not different from controls at baseline or 12-month follow-up (10.2±2.5 vs 9.7±2.1 m/s, p=0.37)	Prospective cohort
Caluwe et al. 2018 [46]	Measurement of PWV in AF	Measurement reliability; effect of cardioversion	cfPWV	N=34; mean age 71±9 years	cfPWV decreased after cardioversion (11.8 to 10.7 m/s); excellent reproducibility (ICC 0.89)	Quasi-experimental study (pre-post within-subject design)
De Vriese et al. 2020 [48]	The Valkyrie study	Modifiability of PWV by anticoagulation in hemodialysis AF patients	cfPWV	N=132; mean age 71±10 years; hemodialysis patients	No difference in PWV changes over 18 months between anticoagulation strategies (p=0.29)	Randomized control trial
Frary et al. 2024 [29]	NT-proBNP and CV risk independent of AS	Composite outcome (HF hospitalization or AF)	cfPWV	N=1,872; mean age 69±8 years	cfPWV not independently associated with composite HF/AF outcome after adjustment (p>0.05)	Prospective cohort
Milan et al. 2020 [41]	PWV and short-term outcomes after volume expansion	NOAF during acute hemodynamic stress	cfPWV	N=41; pilot study	Higher baseline cfPWV associated with adverse outcomes including NOAF	Prospective cohort
Szmigielski et al. 2016 [44]	PWV correlates with aortic atherosclerosis	Aortic atherosclerosis in AF patients undergoing TEE	PWV (type not specified)	N=99; mean age 70.4±11.5 years	Higher PWV associated with advanced aortic atherosclerosis (p<0.0001)	Cross-sectional
Akkaya et al. 2023 [43]	AS and CHA^2^DS^2^-VASc score in AF-related stroke	AF-related stroke	cfPWV	N=30; AF-related stroke patients	Strong correlation between cfPWV and CHA^2^DS^2^-VASc score (r=0.672, p0.001)	Cross-sectional
Shaikh et al. 2016 [28]	Relations of AS and brachial FMD with NOAF: Framingham	Incident AF	cfPWV	N=5,797; median follow-up 7.1 years	cfPWV not associated with AF after full adjustment (p=0.18); augmentation index (HR 1.16, p=0.02), central PP (HR 1.14, p=0.02), and FMD (HR 0.79, p=0.04) were significant	Prospective cohort
Gaczol et al. 2024 [37]	Predicting AF recurrence after pulmonary vein isolation	AF recurrence post-ablation	cfPWV	N=44; paroxysmal AF undergoing PVI	cfPWV did not differ between recurrence and no recurrence groups (p=0.91)	Prospective cohort
Gaczol et al. 2023 [32]	Ventricular-arterial coupling in AF	Established paroxysmal AF	cfPWV	N=108; paroxysmal AF patients	cfPWV elevated vs. controls (p0.05); impaired ventricular-arterial coupling	Cross-sectional

Discussion 

Central vs. Peripheral AS and New-onset vs. Established AF and Why Anatomical and Temporal Determinants Matter

Across multiple cohorts, central PWV predicted incident AF as well as AF-related outcomes, whereas peripheral PWV indices were frequently null or context-dependent [23-27,42,43]. Notably, one prospective cohort demonstrated that baPWV and faPWV (peripheral) were not associated with incident AF despite a significant association with cfPWV (central) [23]. These associations appeared strongest in patients with established CV disease or heightened physiological stress.

As outlined in the introduction, AS promotes AF through adverse VAC, early wave reflection, increased late systolic load, and atrial remodeling. Central PWV captures stiffness of the elastic aorta, where these mechanisms are most relevant, and is supported by data showing that central AS disrupts coupling and impairs LA phasic function [15,16,39,40]. Central AS is more strongly associated with myocardial stress biomarkers and cardiac outcomes than peripheral measures, consistent with the concept that central hemodynamics drive ventricular-vascular coupling abnormalities [49].

By contrast, peripheral PWV reflects muscular AS, which has limited influence on central cardiac loading conditions, explaining its weaker associations. In older adults, peripheral arterial stiffness shows inverse or null associations with CV events, whereas central stiffness remains positively associated, underscoring their distinct prognostic implications [50,51]. Taken together, these findings suggest that central, rather than peripheral, AS more closely links vascular aging to diastolic dysfunction, atrial remodeling, and the development of AF [10,49-51].

Nonlinear and Risk-Dependent Associations Between PWV and AF

Pulse wave velocity demonstrated a U-shaped relationship with incident AF, with both low and high AS associated with increased risk compared with intermediate levels [25]. In other studies, associations between PWV and AF were attenuated or lost after multivariable adjustment for age, blood pressure (BP), and comorbidities, suggesting that PWV may reflect the cumulative burden of demographic and clinical factors that modulate AF risk, such as age, obesity, and hypertension, rather than functioning as an independent risk factor across all populations [28,29]. 

Low PWV can be observed in individuals with frailty, low BP, or autonomic dysfunction, reflecting underlying vulnerability rather than vascular health, which is associated with increased AF risk [25,52,53]. Frailty is independently associated with lower AS in some contexts, yet paradoxically with higher CV risk, suggesting that very low PWV may identify a phenotype characterized by sarcopenia, reduced cardiac output, and autonomic dysregulation [52,54]. In these settings, AF arises from autonomic and electrophysiologic mechanisms rather than pressure-mediated remodeling, allowing AF risk to be high despite low PWV [4].

Elevated PWV marks advanced vascular stiffening associated with atrial remodeling and AF, as described before [12,14]. Consistent with this framework, higher cfPWV is associated with impaired LA reservoir and conduit function independent of BP and LV structure, indicating early atrial myopathy before clinically overt AF [15-17].

Finally, individuals with markedly elevated PWV have substantially increased risks of MI, stroke, and HF, which may lead to death before AF becomes clinically manifest. This competing-risk phenomenon may partially explain age-dependent attenuation of PWV-AF associations despite strong mechanistic links between AS and atrial remodeling [55-57]. In older populations, the high burden of non-CV mortality competes with AF incidence, potentially masking true associations in standard survival analyses that do not account for competing events [56].

AF as a Cause vs. Consequence and the Evidence for Bidirectionality

Higher PWV was found in patients with established AF compared with patients without AF and paroxysmal AF patients [30-32]. In contrast, middle-aged patients presenting with a first episode of AF did not exhibit elevated PWV relative to controls [34]. In this context, longitudinal studies indicate that central hemodynamic indices, such as augmentation index and central pulse pressure, may outperform cfPWV in predicting incident AF in certain populations [27]. These measures more directly reflect pulsatile load and wave reflection abnormalities that contribute to left atrial stretch and the development of an arrhythmogenic substrate [10,12,14].

Collectively, the evidence supports a bidirectional relationship in which AS contributes to AF susceptibility through increased pulsatile atrial load, while sustained AF burden may further impair vascular function via persistent hemodynamic and neurohormonal stress [58-60]. The lack of elevated PWV during first-episode AF likely reflects rhythm-related measurement variability during arrhythmia as well as insufficient time for fixed vascular remodeling to develop [45,46].

AS as a Substrate for Stress-Triggered AF

In patients undergoing cardiac surgery, elevated PWV was strongly associated with POAF, particularly when combined with markers of increased LV filling pressure [39,40]. Similarly, in a model of acute intravascular volume expansion, higher baseline cfPWV predicted short-term adverse outcomes, including NOAF [41].

The association is consistent with a two-hit pathophysiological model, in which chronic vascular remodeling creates latent atrial vulnerability that becomes clinically manifest when acute hemodynamic stress exceeds compensatory capacity. Arterial stiffness contributes to this substrate by promoting atrial stretch, fibrosis, and electrical heterogeneity [12,14]. Acute perioperative stressors, including ischemia-reperfusion injury, inflammation, oxidative stress, and autonomic imbalance, then serve as the second hit, lowering the threshold for AF initiation [60-63]. Within this framework, diastolic dysfunction represents a key hemodynamic expression of the first hit, paralleling chronic AS [58,64,65]. Consequently, acute intravascular volume expansion exacerbates impaired ventricular relaxation and elevates filling pressures, further increasing atrial wall stress and susceptibility to AF during physiological stress [12,14]. This suggests that PWV may identify early atrial myopathy that remains clinically silent until exposed to acute stress, which could become useful for perioperative risk stratification [16,17].

Distinguishing Prognostic From Predictive Roles of PWV in AF

Elevated PWV predicted adverse CV outcomes, including stroke, HF hospitalization, and mortality [35,42-44]. Pulse wave velocity did not meaningfully improve AF risk prediction beyond established clinical risk models, likely reflecting overlap with traditional determinants of arterial stiffness [28,29]. Methodological factors may further contribute to heterogeneity across studies, as PWV measurements obtained during AF show greater variability with two-site techniques, whereas single-site oscillometric methods demonstrate improved reproducibility [45].

Pulse wave velocity predicts outcomes well in patients with established disease but adds little in the general population because it overlaps heavily with age and BP, already included in standard risk scores. Age-related increases in AS closely parallel rises in systolic pressure, making it difficult to disentangle their independent effects once both are included in multivariable models for statistical adjustments [66,67]. Those null findings likely reflect collinearity rather than a lack of biological importance [68,69]. This issue can be further compounded by overadjustment for downstream consequences such as HF or LV hypertrophy.

Age-dependent effects further clarify this distinction. Meta-analytic data indicate that PWV provides greater incremental predictive value in younger and intermediate-risk individuals, in whom BP is a less reliable surrogate for vascular stiffness [70]. In older populations, systolic BP more closely reflects aortic stiffening, limiting the added value of PWV despite its continued pathophysiologic relevance. Accordingly, PWV is best viewed not as a standalone predictor but as an integrative marker of hypertension (HTN)-mediated organ damage that captures vascular, hemodynamic, and metabolic risk within a single measure [9,10].

Context-Dependent Prognostic Value and Modifiability of PWV in AF

A higher aPWV was associated with increased recurrence frequency in medically managed patients with paroxysmal AF, with a defined stiffness threshold (aPWV >10.0 m/s) predicting recurrence burden [36]. Atrial fibrillation inducibility during electrophysiology testing was unrelated to PWV and instead associated with endothelial dysfunction [38]. No significant change in PWV was observed across anticoagulation strategies in patients with end-stage renal disease (ESRD) and AF, indicating limited modifiability in advanced vascular disease states [48]. Pulse wave velocity did not predict recurrence following pulmonary vein isolation, and AS measures did not differ between patients with and without post-ablation AF recurrence [37].

In medically treated patients, elevated AS remains associated with AF recurrence because pharmacologic therapy does not correct the underlying ventricular-vascular atrial coupling abnormalities that drive progressive atrial stretch, fibrosis, and electrical substrate maturation over time, allowing PWV to retain prognostic relevance in this setting [14,58]. In contrast, after pulmonary vein isolation, AF recurrence is driven by local atrial structural factors (including pulmonary vein reconnection) and procedural durability rather than by systemic vascular stiffness, explaining the loss of PWV prognostic value in the post-ablation setting [71-74].

Similarly, AF inducibility reflects acute endothelial and autonomic disturbances driven by oxidative stress and inflammation rather than chronic AS [38,59,75]. The lack of PWV improvement in patients with AF and ESRD further illustrates a pathophysiological ceiling, as extensive medial calcification, fibrosis, and disordered mineral metabolism produce largely irreversible AS that is resistant to hemodynamic or pharmacologic modulation [48,76]. In this setting, PWV reflects fixed vascular damage rather than a modifiable physiological state [10,12]. Overall, the association between PWV and AF outcomes appears to be determined by chronic vascular disease rather than acute rhythm or hemodynamic changes.

However, not all findings align with a purely chronic substrate model, as some evidence demonstrates the opposite, with acute modulation of PWV and diminished responsiveness in advanced disease states. For example, pharmacologic modulation was also observed, with PWV reduction after switching from warfarin to rivaroxaban [47]. In another study, cfPWV decreased modestly following successful cardioversion to sinus rhythm while demonstrating excellent reproducibility, consistent with partial reversibility related to acute hemodynamic normalization rather than structural vascular change [46]. 

The heterogeneous modifiability of PWV reflects its dual nature as both a structural indicator and a dynamic hemodynamic marker influenced by extrinsic determinants such as BP, rhythm regularity, and metabolic state [10,12]. This duality explains why PWV may decrease with hemodynamic normalization, as rhythm regularization reduces beat-to-beat variability in stroke volume and arterial loading that mechanically stresses the arterial wall while remaining resistant to change once fixed structural remodeling predominates [12,77]. Accordingly, the modest reduction in PWV observed after cardioversion is best interpreted as a functional effect: restoration of sinus rhythm improves cardiac efficiency, reduces BP variability, and enhances peripheral vasodilatory reserve, transiently lowering measured PWV without altering arterial wall structure [46,58].

The reduction observed after transition from warfarin to rivaroxaban may reflect rivaroxaban's ability to attenuate endothelial inflammation, reduce pro-inflammatory extracellular vesicles, and improve endothelial function, each of which influences vascular tone and smooth muscle reactivity [47,78]. By contrast, vitamin K antagonism has been linked to vascular calcification, providing a potential mechanism by which long-term warfarin exposure may exacerbate arterial calcification and stiffness, further enhancing the comparative benefits of switching to rivaroxaban [79-81].

Strengths, Limitations, and Future Research

This scoping review provides a comprehensive synthesis of PWV across the full clinical spectrum of AF, including incident AF, established subtypes, POAF, recurrence, treatment response, and downstream outcomes. This review extends beyond AF occurrences alone to investigate disease burden, progression, and prognosis. A key strength is the inclusion of multiple PWV modalities and vascular territories, allowing direct comparison between central and peripheral measures. The consistent predominance of central PWV associations strengthens the biological plausibility linking AS, VAC, diastolic dysfunction, and atrial remodeling. Findings from large cohort studies were interpreted in the context of underlying pathophysiology, helping to explain inconsistent or nonlinear associations across studies.

Several limitations merit consideration. Most included studies were observational and cross-sectional, limiting causal inference and making it unclear whether AS precedes or results from AF. There was also substantial variability in PWV measurement techniques and vascular segments assessed, limiting comparability across studies. Measurement during AF introduces additional variability due to rhythm irregularity, and the absence of standardized AF-specific PWV protocols likely contributed to inconsistent findings. Residual confounding remains a concern, particularly for factors such as autonomic dysfunction, inflammation, endothelial function, and genetic susceptibility, which were inconsistently measured. Conversely, adjustment for downstream variables such as BP, HF, or LV hypertrophy may have led to overadjustment and attenuation of true associations. Finally, variability in PWV thresholds and outcome definitions limited assessment of dose-response relationships and identification of clinically meaningful cutoff values.

Future research should prioritize longitudinal studies with serial assessment of AS and atrial structure and function to clarify temporal relationships and disease progression, particularly transitions between AF subtypes. Standardization of PWV measurement in AF populations, including guidance on rhythm documentation, averaging strategies, and preferred vascular territories, is needed to improve reproducibility. Interventional studies may help clarify whether PWV represents a modifiable contributor to AF risk or primarily a marker of cumulative vascular disease. Finally, evaluating PWV in targeted clinical contexts, such as perioperative AF, early-stage disease, and younger or intermediate-risk populations, may help define its role in risk stratification when integrated with echocardiographic and clinical markers of atrial cardiomyopathy.

## Conclusions

Overall, the existing literature supports a consistent association between elevated PWV and AF, particularly in relation to atrial remodeling, adverse outcomes, and perioperative AF. Elevated PWV likely reflects cumulative vascular remodeling and hemodynamic stress that may contribute to atrial vulnerability, but the predominance of observational designs limits causal inference regarding directionality. Across studies, PWV appears most informative as a marker of disease severity, physiologic stress, and prognosis in established or high-risk contexts rather than as a standalone predictor of AF onset in general populations. Future work should prioritize standardized, AF-specific PWV protocols and longitudinal designs to clarify temporality and to test whether PWV improves prognostic stratification and perioperative risk assessment when integrated with echocardiographic and clinical markers of atrial cardiomyopathy.

## Appendices

Appendix A

Featured below in Tables 3-6 are the full search strings used in this review for each of the four databases.

**Table 3:** Search strings used in EMBASE

No.	Search string terms	Results
#1	atrial fibrillation'/exp	2,46,511
#2	atrium fibrillation*':ab,ti,kw OR 'auricular fibrillation*':ab,ti,kw OR 'cardiac atrial fibrillation*':ab,ti,kw OR 'cardiac atrium fibrillation*':ab,ti,kw OR 'heart atrial fibrillation*':ab,ti,kw OR 'heart atrium fibrillation*':ab,ti,kw OR 'non-valvular atrial fibrillation*':ab,ti,kw OR 'nonvalvular atrial fibrillation*':ab,ti,kw OR 'valvular atrial fibrillation*':ab,ti,kw OR 'persistent atrial fibrillation*':ab,ti,kw OR 'familial atrial fibrillation*':ab,ti,kw OR 'paroxysmal atrial fibrillation*':ab,ti,kw OR 'fibrillation*, heart atrium':ab,ti,kw OR 'heart fibrillation* atrium':ab,ti,kw	27,154
#3	#1 OR #2	2,47,908
#4	pulse wave velocity'/exp	7,709
#5	pwv, pulse wave velocit*':ab,ti,kw OR 'pwv':ab,ti,kw OR 'pulse wave velocit*':ab,ti,kw OR 'aort* pulse wave velocit*':ab,ti,kw OR 'aort* pwv':ab,ti,kw OR 'aort* pwv, pulse wave velocit*':ab,ti,kw OR 'pulse wave velocit* of aorta':ab,ti,kw OR 'pwv of aorta':ab,ti,kw OR 'apwv':ab,ti,kw OR 'arter*l pulse wave velocit*':ab,ti,kw OR 'arter* pwv':ab,ti,kw OR 'arter* pwv, pulse wave velocit*':ab,ti,kw OR 'arter* wave velocit*':ab,ti,kw OR 'arter* pulse velocit*':ab,ti,kw OR 'brachial-ankle pulse wave velocit*':ab,ti,kw OR 'brachial-ankle pwv':ab,ti,kw OR 'bapwv, brachial-ankle pulse wave velocit*':ab,ti,kw OR 'bapwv':ab,ti,kw OR 'brachial-ankle pulse velocit*':ab,ti,kw OR 'brachial-ankle wave velocit*':ab,ti,kw OR 'carotid-femoral pulse wave velocit*':ab,ti,kw OR 'carotid-femoral pwv':ab,ti,kw OR 'cfpwv, carotid-femoral pulse wave velocit*':ab,ti,kw OR 'cfpwv':ab,ti,kw OR 'carotid-femoral pulse velocit*':ab,ti,kw OR 'carotid-femoral wave velocit*':ab,ti,kw OR 'carotid-radial pulse wave velocit*':ab,ti,kw OR 'carotid-radial pwv':ab,ti,kw OR 'crpwv, carotid-radial pulse wave velocit*':ab,ti,kw OR 'crpwv':ab,ti,kw OR 'carotid-radial wave velocit*':ab,ti,kw OR 'carotid-radial pulse velocit*':ab,ti,kw OR 'pulse wave analys*':ab,ti,kw	26,780
#6	#4 OR #5	27,868
#7	#3 AND #6	390
#8	#7 AND (2014:py OR 2015:py OR 2016:py OR 2017:py OR 2018:py OR 2019:py OR 2020:py OR 2021:py OR 2022:py OR 2023:py OR 2024:py)	325

**Table 4:** Search strings used in Ovid MEDLINE

No.	Search string terms	Results
#1	exp Atrial Fibrillation/	77,710
#2	('atrium fibrillation*' or 'auricular fibrillation*' or 'cardiac atrial fibrillation*' or 'cardiac atrium fibrillation*' or 'heart atrial fibrillation*' or 'heart atrium fibrillation*' or 'non-valvular atrial fibrillation*' or 'nonvalvular atrial fibrillation*' or 'valvular atrial fibrillation*' or 'persistent atrial fibrillation*' or 'familial atrial fibrillation*' or 'paroxysmal atrial fibrillation*' or 'fibrillation*, heart atrium' or 'heart fibrillation* atrium').ti, ab, kf.	15,665
#3	1 or 2	81,561
#4	*Pulse Wave Analysis/ or *Carotid-Femoral Pulse Wave Velocity/	2,147
#5	('pwv' or 'pulse wave velocit*' or 'aort* pulse wave velocit*' or 'aort* pwv' or 'pulse wave velocit* of aorta' or 'pwv of aorta' or 'apwv' or 'arter*l pulse wave velocit*' or 'arter* pwv' or 'arter* wave velocit*' or 'arter* pulse velocit*' or 'brachial-ankle pulse wave velocit*' or 'brachial-ankle pwv' or 'bapwv' or 'brachial-ankle pulse velocit*' or 'brachial-ankle wave velocit*' or 'carotid-femoral pulse wave velocit*' or 'carotid-femoral pwv' or 'cfpwv' or 'carotid-femoral pulse velocit*' or 'carotid-femoral wave velocit*' or 'carotid-radial pulse wave velocit*' or 'carotid-radial pwv' or 'crpwv' or 'carotid-radial wave velocit*' or 'carotid-radial pulse velocit*' or 'pulse wave analys*').ti, ab, kf.	14,140
#6	4 or 5	14,659
#7	3 and 6	49
#8	limit 7 to yr="2014 - 2024"	36

**Table 5:** Search strings used in Web of Science

No.	Search string terms	Results
#1	TS=("atrial fibrillation*" OR "atrium fibrillation*" OR "auricular fibrillation*" OR "cardiac atrial fibrillation*" OR "cardiac atrium fibrillation*" OR "heart atrial fibrillation*" OR "heart atrium fibrillation*" OR "non-valvular atrial fibrillation*" OR "nonvalvular atrial fibrillation*" OR "valvular atrial fibrillation*" OR "persistent atrial fibrillation*" OR "familial atrial fibrillation*" OR "paroxysmal atrial fibrillation*" OR "fibrillation*, heart atrium" OR "heart fibrillation* atrium" )	1,38,305
#2	TS=("pulse wave velocity" OR "pwv" OR "pwv (pulse wave velocit*)" OR "pulse wave velocit*" OR "aort* pulse wave velocit*" OR "aort* pwv" OR "aort* pwv (pulse wave velocit*)" OR "pulse wave velocit* of aorta" OR "pwv of aorta" OR "apwv" OR "arter*l pulse wave velocit*" OR "arter* pwv" OR "arter* pwv (pulse wave velocit*)" OR "arter* wave velocit*" OR "arter* pulse velocit*" OR "brachial-ankle pulse wave velocit*" OR "brachial-ankle pwv" OR "bapwv (brachial-ankle pulse wave velocit*)" OR "bapwv" OR "brachial-ankle pulse velocit*" OR "brachial-ankle wave velocit*" OR "carotid-femoral pulse wave velocit*" OR "carotid-femoral pwv" OR "cfpwv (carotid-femoral pulse wave velocit*)" OR "cfpwv" OR "carotid-femoral pulse velocit*" OR "carotid-femoral wave velocit*" OR "carotid-radial pulse wave velocit*" OR "carotid-radial pwv" OR "crpwv (carotid-radial pulse wave velocit*)" OR "crpwv" OR "carotid-radial wave velocit*" OR "carotid-radial pulse velocit*" OR "pulse wave analys*")	19,542
#3	#2 AND #1	101

**Table 6:** Search strings used in CINAHL

No.	Search string terms	Results
#1	(MH "Atrial Fibrillation+")	30,603
#2	TI ( ("atrial fibrillation*" OR "atrium fibrillation*" OR "auricular fibrillation*" OR "cardiac atrial fibrillation*" OR "cardiac atrium fibrillation*" OR "heart atrial fibrillation*" OR "heart atrium fibrillation*" OR "non-valvular atrial fibrillation*" OR "nonvalvular atrial fibrillation*" OR "valvular atrial fibrillation*" OR "persistent atrial fibrillation*" OR "familial atrial fibrillation*" OR "paroxysmal atrial fibrillation*" OR "fibrillation*, heart atrium" OR "heart fibrillation* atrium" ) ) OR AB ( ("atrial fibrillation*" OR "atrium fibrillation*" OR "auricular fibrillation*" OR "cardiac atrial fibrillation*" OR "cardiac atrium fibrillation*" OR "heart atrial fibrillation*" OR "heart atrium fibrillation*" OR "non-valvular atrial fibrillation*" OR "nonvalvular atrial fibrillation*" OR "valvular atrial fibrillation*" OR "persistent atrial fibrillation*" OR "familial atrial fibrillation*" OR "paroxysmal atrial fibrillation*" OR "fibrillation*, heart atrium" OR "heart fibrillation* atrium" ) )	38,743
#3	S1 OR S2	45,994
#4	(MH "Pulse Wave Velocity+")	553
#5	TI ( ("pulse wave velocity" OR "pwv" OR "pwv (pulse wave velocit*)" OR "pulse wave velocit*" OR "aort* pulse wave velocit*" OR "aort* pwv" OR "aort* pwv (pulse wave velocit*)" OR "pulse wave velocit* of aorta" OR "pwv of aorta" OR "apwv" OR "arter*l pulse wave velocit*" OR "arter* pwv" OR "arter* pwv (pulse wave velocit*)" OR "arter* wave velocit*" OR "arter* pulse velocit*" OR "brachial-ankle pulse wave velocit*" OR "brachial-ankle pwv" OR "bapwv (brachial-ankle pulse wave velocit*)" OR "bapwv" OR "brachial-ankle pulse velocit*" OR "brachial-ankle wave velocit*" OR "carotid-femoral pulse wave velocit*" OR "carotid-femoral pwv" OR "cfpwv (carotid-femoral pulse wave velocit*)" OR "cfpwv" OR "carotid-femoral pulse velocit*" OR "carotid-femoral wave velocit*" OR "carotid-radial pulse wave velocit*" OR "carotid-radial pwv" OR "crpwv (carotid-radial pulse wave velocit*)" OR "crpwv" OR "carotid-radial wave velocit*" OR "carotid-radial pulse velocit*" OR "pulse wave analys*") ) OR AB ( ("pulse wave velocity" OR "pwv" OR "pwv (pulse wave velocit*)" OR "pulse wave velocit*" OR "aort* pulse wave velocit*" OR "aort* pwv" OR "aort* pwv (pulse wave velocit*)" OR "pulse wave velocit* of aorta" OR "pwv of aorta" OR "apwv" OR "arter*l pulse wave velocit*" OR "arter* pwv" OR "arter* pwv (pulse wave velocit*)" OR "arter* wave velocit*" OR "arter* pulse velocit*" OR "brachial-ankle pulse wave velocit*" OR "brachial-ankle pwv" OR "bapwv (brachial-ankle pulse wave velocit*)" OR "bapwv" OR "brachial-ankle pulse velocit*" OR "brachial-ankle wave velocit*" OR "carotid-femoral pulse wave velocit*" OR "carotid-femoral pwv" OR "cfpwv (carotid-femoral pulse wave velocit*)" OR "cfpwv" OR "carotid-femoral pulse velocit*" OR "carotid-femoral wave velocit*" OR "carotid-radial pulse wave velocit*" OR "carotid-radial pwv" OR "crpwv (carotid-radial pulse wave velocit*)" OR "crpwv" OR "carotid-radial wave velocit*" OR "carotid-radial pulse velocit*" OR "pulse wave analys*") )	4,091
#6	S4 OR S5	4182
#7	S3 AND S6 With Limiters - Publication Date: 20140101-20241231	28
